# Polysialylation of NCAM Characterizes the Proliferation Period of Contractile Elements during Postnatal Development of the Epididymis

**DOI:** 10.1371/journal.pone.0123960

**Published:** 2015-03-30

**Authors:** Peter Simon, Caroline Feuerstacke, Miriam Kaese, Farhan Saboor, Ralf Middendorff, Sebastian P. Galuska

**Affiliations:** 1 Institute of Biochemistry, Medical Faculty, Justus-Liebig-University, Friedrichstr. 24, 35392, Giessen, Germany; 2 Department of Anatomy and Cell Biology, Medical Faculty, Justus-Liebig-University, Aulweg 123, 35385, Giessen, Germany; National Cancer Institute, UNITED STATES

## Abstract

Polysialic acid (polySia) attached to the neural cell adhesion molecule (NCAM) regulates *inter alia* the proliferation and differentiation via the interactions with neurotrophins. Since in postnatal epididymis neurotrophins and their receptors like the Low-Affinity Nerve Growth Factor Receptor p75 and TrK B receptor are expressed, we wanted to analyze if the polysialylation of NCAM is also involved during the development of the epididymis. To this end, we monitored the developmental changes in the expression of the polysialyltransferases and NCAM polysialylation using murine epididymis at different time points during postnatal development. Our results revealed that during postnatal development of the epididymis both polysialyltransferases, ST8SiaII and ST8SiaIV, were expressed and that the expression levels dropped with increasing age. In agreement with the expression levels of the polysialyltransferases the highest content of polysialylated NCAM was present during the first 10 days after birth. Interestingly, proliferating smooth muscle cell populations prevalently expressed polysialylated NCAM. Furthermore, we observed that inverse to the decrease in polysialylation of smooth muscle cells a strong up-regulation of collagen takes place suggesting a functional relationship since collagen was recently described to induce the turnover of polysialylated NCAM via an induction of endocytosis *in cellulo*. The same time course of polySia and collagen synthesis was also observed in other regions of the male reproductive system e.g. vas deferens and tunica albuginea (testis). Together, we identified a spatio-temporal expression pattern of polySia-NCAM characterized by high proliferation rate of smooth muscle cells and low collagen content.

## Introduction

In mammals sialic acid polymers are present as posttranslational modification of distinct glycoproteins, whereupon the neuronal cell adhesion molecule NCAM represents the major carrier of this strongly anionic charged carbohydrate epitope [1–4]. Two polysialyltransferases, ST8SiaII and ST8SiaIV, are able to synthesize this unique carbohydrate structure composed of α2,8-linked sialic acid residues. Variations in the gene dosages of these enzymes resulted in alterations in the total amount as well as in the degree of polymerization of polySia on N-glycans of NCAM [5–7]. Three major isoforms of NCAM are expressed and can be a target for ST8SiaII and ST8SiaIV. These isoforms differ in their membrane attachment or cytoplasmic tails and are termed NCAM-180, NCAM-140 (trans-membrane isoforms) and NCAM-120 (glycosylphosphatidyinositol-anchored isoform) according to their apparent molecular weights [8].

Interestingly, the inactivation of both polysialyltransferases leads to a lethal phenotype [9].

Different physiological functions of polysialylated NCAM (polySia-NCAM) are discussed depending e.g. on the expressing cell types and stage of differentiation [10–15]. For example, in the respiratory system polysialylated NCAM is released under inflammatory conditions and polysialic acid (polySia) is proposed to counteract the cytotoxic properties of extracellular histones [16], which are accumulated during the formation of neutrophil extracellular traps (NET) [17,18]. A comparable function takes probably place in the female reproductive tract after insemination. Also here neutrophils generate NET and sperms are trapped in this network of DNA and histones [19]. PolySia in semen may contribute to increase the number of vital sperms via its ability to bind and inactivate histones [4].

In testis, however, polysialylation of NCAM is discussed in another context. For an efficient proliferation of the spermatogonial stem cell population, Sertoli cells have to separate these cells after each cell division [20]. Since polySia is described to counteract cell adhesion via its polyanionic characteristics we suggested that sialic acid polymers attached to NCAM may support the detachment of the cell-cell contact between spermatogonia after mitosis [21]. Furthermore, polySia can bind several growth factors and neurotrophins like basic fibroblast growth factor (bFGF), neurotrophin 3 (NT-3) and brain derived nerve growth factors (BDNF) [2]. For example the accumulation of BDNF on polySia chains increases its impact on growth or/and survival of neuroblastoma cells *in cellulo* [22]. In contrast, a complex of polySia and bFGF leads to inhibition of bFGF-induced cell growth. These different interplays may also take part during testis development. The polysialylated form of NCAM is present during early testicular organogenesis on the cellular surface of Sertoli cells in addition to spermatogonia [21] and also BDNF and other neurotrophins were described to be expressed during testis development supporting seminiferous cord formation and the survival of germ cells [23].

In a similar way, polySia may contribute to the development of a second part in the male reproductive system, the epididymis. Receptors for BDNF and NT-3 and other neurotrophins are reported to be expressed in α smooth muscle isoactin (SMA) positive mesenchymal cells during postnatal development of epididymis [24] and an isoform of NCAM, not characterized in detail by these authors, was observed in postnatal epididymis [25]. In order to study a possible functional role of polySia in this context, we examined murine epididymal tissue during postnatal development. Our results showing a time depending expression profile of polysialylated NCAM on SMA positive cells during a time window characterized by a high proliferation rate and low collagen expression.

## Material and Methods

### Materials

NCAM-specific monoclonal antibody (mAb) H28 [26] and polySia-specific mAb 735 as well as inactive and active endoneuraminidase (endoN) were purified as described previously [27,28]. The anti-NCAM mAb H28 recognized the extracellular domain of all three isoforms of NCAM. In addition monoclonal anti-alpha smooth muscle Actin antibody 1A4 (Abcam Cambridge, UK) as well as anti-PCNA antibody (Epitomics, Burlingame, U.S.A.) were used according to manufacturers’ introductions. All reagents used were of analytical grade.

### Animals

Epididymal tissues were obtained from mice (black 6) of different ages (postnatal day 1, 4, 7, 10, 15, 20 and 25) housed in the animal facility of Justus-Liebig-University Giessen. In addition testis and vas deferens were collected. Housing, animal care and all procedures were conducted according to the guidelines for animal care and approved by the committee for laboratory animals of Justus-Liebig-University Giessen, case number A38/2011_V54-19c2015(1)GI20/23 and A29/2009_V54-19c20/15cGI20/23. Animals were scarified by manual cervical dislocation.

### SDS-PAGE and Western Blotting

Whole postnatal mouse epidydimidis samples were homogenized in lysis buffer (50 mM Tris/HCL at pH 7.5, 150mM NaCl, 5mM EDTA, 1% Triton X-100, 0,5% sodium-deoxychlorate, 2mM phenylmethylsulfonylfluoride (PMSF), 1mM Aprotinin, 1mM Leupeptin) for 10 min at room temperature. Total protein lysates concentrations were measured by Pierce BCA Protein Assay Kit (Thermo Scientific, Rockford, IL USA) according to manufacturers’ introductions. For negative control polySia was degraded by EndoN digest (1 μg/ml) for 4 h at 37°C. Before separation by 8% SDS-PAGE and subsequent transference onto a PVDF membrane, lysates were reduced by Laemmli Sample Buffer (Bio Rad laboratories, Neuberg, Germany) according to manufacturers introductions. Bound primary antibodies (Anti-polySia and Anti-NCAM H28) were detected by HRP-conjugated antibodies (Dako, Hamburg, Germany) and chemiluminescence SuperSignal kit (Thermo Fisher, Kehl, Germany).

### Immunhistochemistry

Paraffin embedded testis, vas deferens and epididymis tissue sections were cut into 5 μm serial sections. After rehydration in xylene and a following descending ethanol series, sections were incubated with blocking solution for 5 min, followed by incubation with the primary antibody mAb 735 (10 μg/ml PBS/2% (w/v)), anti-SMA antibody (Abcam Laboratories, Cambridge, UK) (0.66 μg/ml) PBS/2% (w/v) or anti-PCNA antibody (Epitomics, Burlingame U.S.A.) (at a 1:500 dilution) PBS/2% (w/v) overnight at 4°C. For negative control of polySia immunostaining, tissue sections were pretreated with endoN (3 μg/ml in PBS/0.1% BSA) overnight at 37°C. For staining the Envision^+^ System HRP Kit (Dako, Hamburg, Germany) was used. The stained sections were counterstained with hemalaun (Roth, Karlsruhe, Germany). For the visualization of collagen fibers Heidenhain's AZAN trichrome staining was performed as described previously [29]. Staining proceedings and incubation time of all experiments were strictly identic to ensure standardized results for comparison. All images were taken with a Zeiss Axioplan 2 imaging microscope and Axiovision LE software.

### mRNA analyses of ST8SiaII and ST8SiaIV

Total cellular RNA was prepared from mouse epididymis at the time of explantation by RNAeasy micro kit (Qiagen, Hilden, Germany). Subsequently, first strand cDNA was generated by iScript cDNA Synthesis Kit (Bio Rad Laboratories). Intron spanning primers were used to amplify ST8Sia II, ST8Sia IV and β-Actin targets using SYBR Green Real-Time PCR Master Mixes (Invitrogen life technologies) according to manufacturers’ introductions. Primers used in quantitative PCR expression analysis:

ST8SiaII for: AGACACAACCAGACGCTCTCTCT,

ST8SiaII rev: GAATAATGTCTCCAGGCTTCAGG;

ST8SiaIV for: CAAAAGAAATAGCCAGAACTGAGG,

ST8SiaIV rev: CCTTCCGGATGATTTTATCAGAG;

β-Actin for: CCATCATGAAGTGTGACGTTGA,

β-Actin rev: CATCGTACTCCTGCTTGCTGAT.

cDNA samples were denatured at 95°C for 3:30 min, followed by 35 cycles of each 20 s at 95°C, 20 s at 60°C and 20 s at 72°C. For melting curve analysis PCR samples were slowly heated from 72°C to 95°C and stored at 20°C. Amplified products were separated by gel electrophoresis using 2% agarose gel for quality control. In addition, amplification efficiency was determined by analyzing the slope of a CT/log (template concentration) plot and primer efficiencies of 100% +/- 5% were accepted for normalization. Emitted fluorescence was detected online using SYBR green real-time PCR system (Bio Rad, iCycler iQ Real-Time PCR Detection System).

### Statistics

Data were analyzed by One-way ANOVA analysis followed by Dunnett’s test to determine statistical significance of the differences using GraphPad Prism version 6.00, (GraphPad Software, La Jolla, California). Significance values are *P<0.05, **P<0.01, ***P<0.001 and ns for non-significant (P>0.05).

## Results

### Polysialylation profiles during postnatal development of epididymis

**Fig 1:**
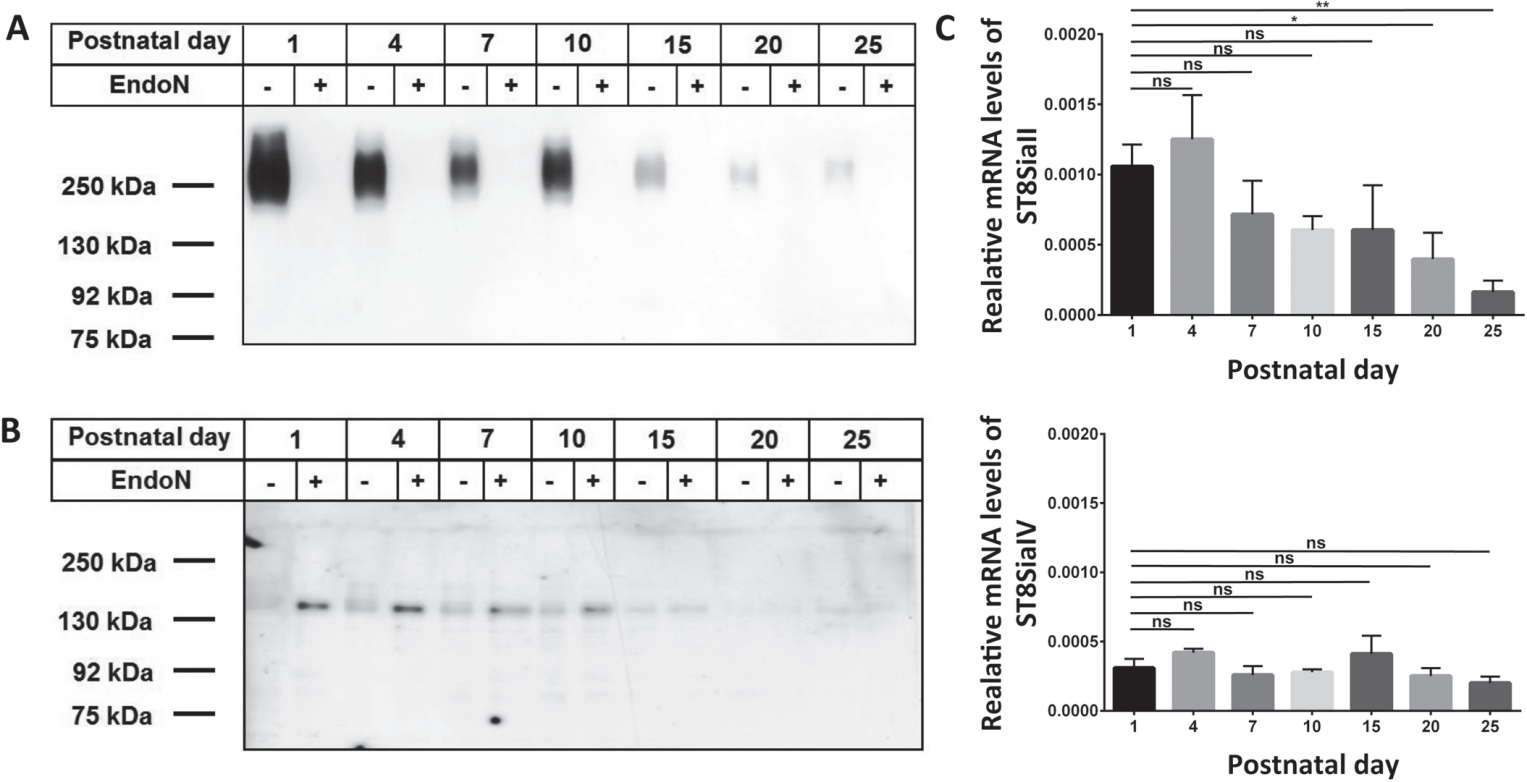
Polysialylation status and expression levels of ST8SiaII and ST8SiaIV during postnatal development of epididymis. Postnatal epididymis homogenates were separated by SDS-PAGE for Western blotting using 0.3 *μ*g protein per lane with or without endoN pretreatment. Immunostaining was performed with an (A) anti-polySia mAb and (B) anti-NCAM mAb H28. The expected molecular weight of the polysialylated form of NCAM ranged between 120 and 300 kDa. Apparent molecular weights of standard proteins are indicated in kDa. (C) mRNA expression levels of ST8SiaII and ST8SiaIV in postnatal epididymis were determined by quantitative real time PCR. β-Actin was used as standard housekeeping gene to calculate the relative mRNA values of ST8SiaII and ST8SiaIV (mRNA β-Actin / mRNA polysialyltransferases). Values represent means ± SEM (standard error of the mean) of 3 independent experiments. *, P<0.05; **, P<0.01; ns = non-significant).

Nerve growth factors like BDNF are discussed to influence the differentiation of epididymal cells since SMA-positive cells express receptors for neurotrophins such as the Low-Affinity Nerve Growth Factor Receptor p75 and TrK B receptor during the postnatal development of the epididymis [24]. Since polySia attached to NCAM influences the properties of neurotrophins [22,30], we analyzed the postnatal expression pattern of polysialylated NCAM in the postnatal epididymis. Tissues were isolated at subsequent days after birth (P1 to P25) and for each time point equal amounts of protein homogenates were subjected to SDS-PAGE and Western blotting against polySia (Fig 1A). For negative control polySia was degraded by endoN. The achieved immune staining demonstrated that polySia was highly expressed during the first days of postnatal development. After P10, however, a down regulation of polySia occurred.

In addition, Western blotting against NCAM was performed (Fig 1B). Since polySia may inhibit the binding of antibodies in the same way as the homophilic NCAM-NCAM binding only the un- and/or de-polysialylated protein backbone of NCAM exhibited strong immune signals. In line with the anti-polySia Western blots obtained immune staining against the polysialylated and the de-polysialylated form of NCAM revealed that up to P10 nearly the complete NCAM-140 pool was polysialylated. NCAM-120 and -180 were not detectable. Thereafter, the signal intensities of NCAM-140 in untreated homogenates were comparable with the intensities of endoN treated samples. Consequently, polySia attached to NCAM-140 is mainly build in the epididymis during the first 10 days of postnatal development.

To determine which polysialyltransferase contribute to the generation of polySia chains in the postnatal epididymis we estimated the transcript levels of ST8SiaII and ST8SiaIV. As shown in Fig 1C both polysialyltransferases were expressed in the neonatal epididymis. However, 3.5 fold higher mRNA values were determined for ST8SiaII. During the postnatal period and puberty the expression level of ST8SiaII was significantly reduced. The mRNA level of ST8SiaIV, however, decreases only slightly during postnatal development and the calculated differences were not significant. Thus, ST8SiaII was the predominant polysialyltransferase in perinatal mouse epididymis whereas in adult animals the expression of ST8SiaIV was more pronounced as already described by us in Simon *et al*. [4].

### Polysialylated NCAM is predominately express by smooth muscle cells during postnatal development of epididymis

**Fig 2:**
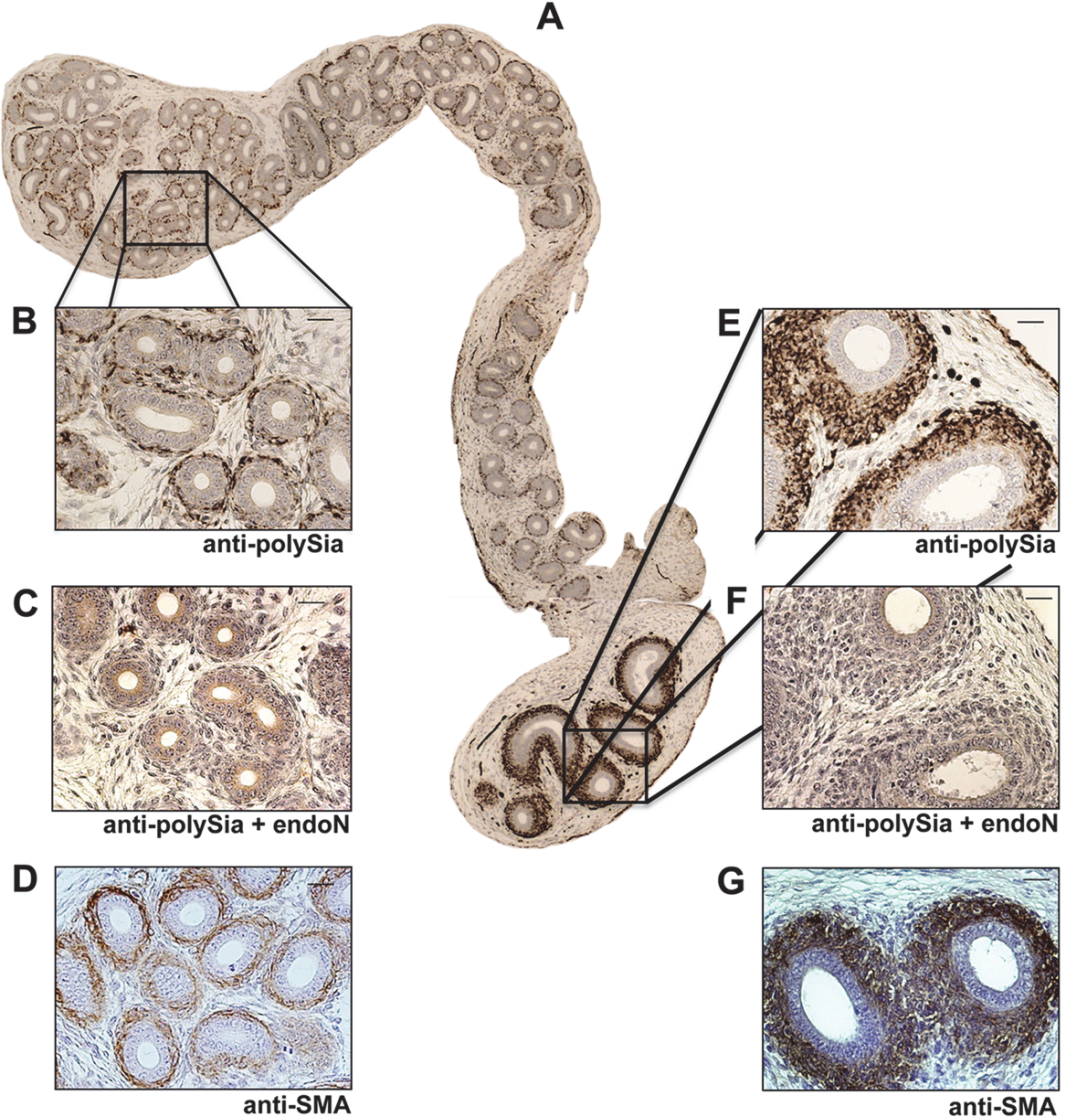
Immunohistological localization of polySia in epididymal tissue of 4-day-old mice. (A-G) Paraffin-embedded serial epididymis sections. Whole longitudinal epididymis of mice were stained with a mAb against polySia (A,B,E,). For negative control, tissue sections were pretreated with endoN to degrade polySia (C,G). For identification of smooth muscle cells mAB against smooth muscle actin (D,G) were used. Tissues were counterstained with Haemalaun. Scale bars representing 20 μm.

**Fig 3:**
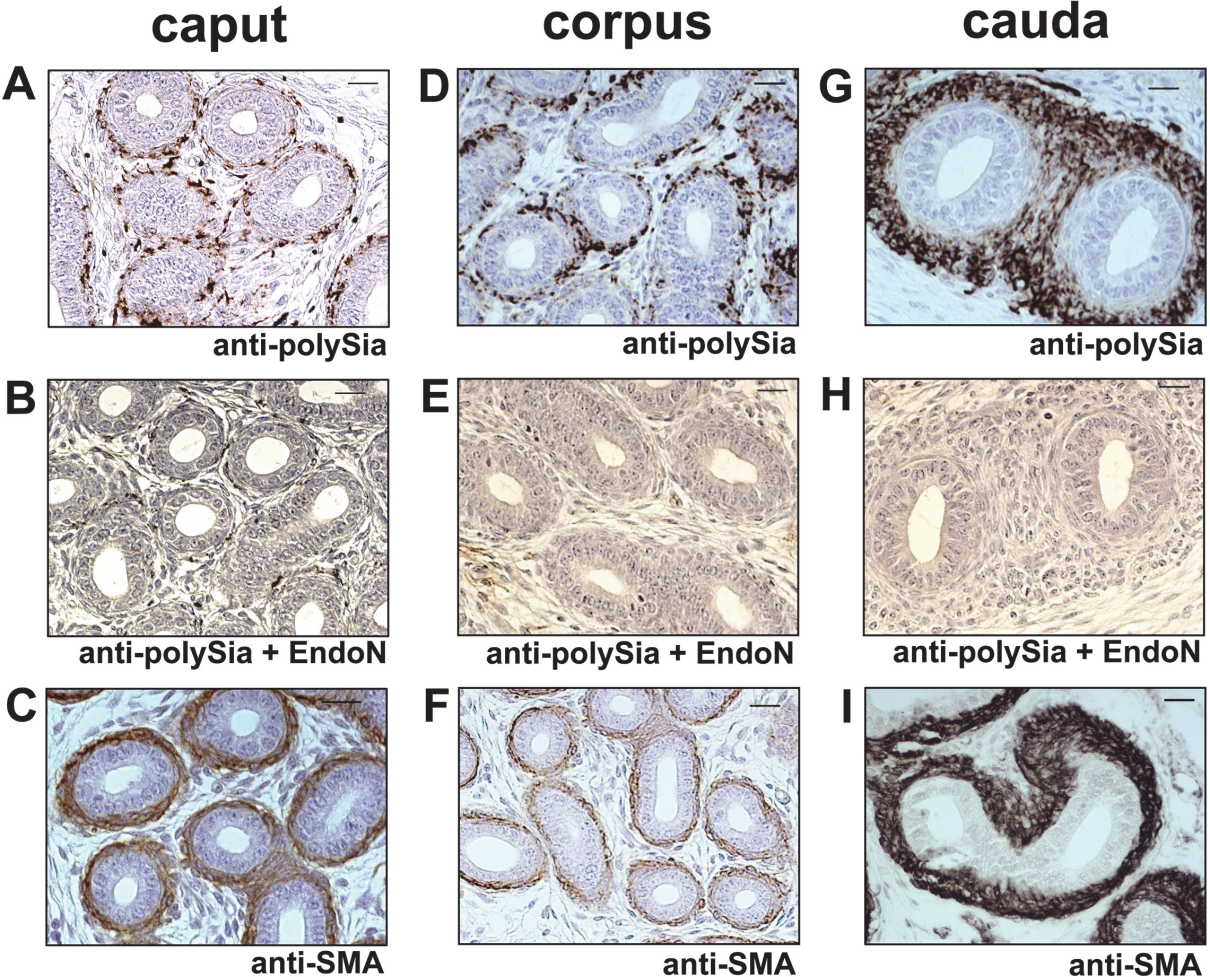
Immunohistological localization of polySia in epididymal tissue of 10-day-old mice. (A)-(F) Paraffin-embedded serial sections of caput, corpus and cauda epididymis. PolySia^+^ cells were identified with a mAb against polySia (A,D,G). For negative control, tissue sections were pretreated with endoN to degrade polySia (B,E,H). For identification of smooth muscle cells a mAb against smooth muscle actin (C,F,I) were used. Sections were counterstained with Haemalaun. Scale bars representing 20 μm.

To investigate which cell types are polysialylated postnatally, we performed immunohistochemical analyses using longitudinal epididymis sections of 4-day-old mice (Fig 2). In all parts of the epididymis polySia was localized to smooth muscle cells surrounding the epithelial layer of the epididymal duct (Fig 2A). In addition, scattered epithelia cells showed a polySia positive signal (data not shown). Specificity of polySia immunostaining is visible by pre-treatment of the sections with EndoN prior use of the antibody as shown by higher magnification of caput and cauda regions (Fig 2B, 2C, 2E and 2F). SMA staining procedures verified the smooth muscle cell phenotype (Fig 2D and 2G). PolySia was also specifically found in smooth muscle cells of the epididymal duct when investigating 10-day-old mice (Fig 3).

**Fig 4:**
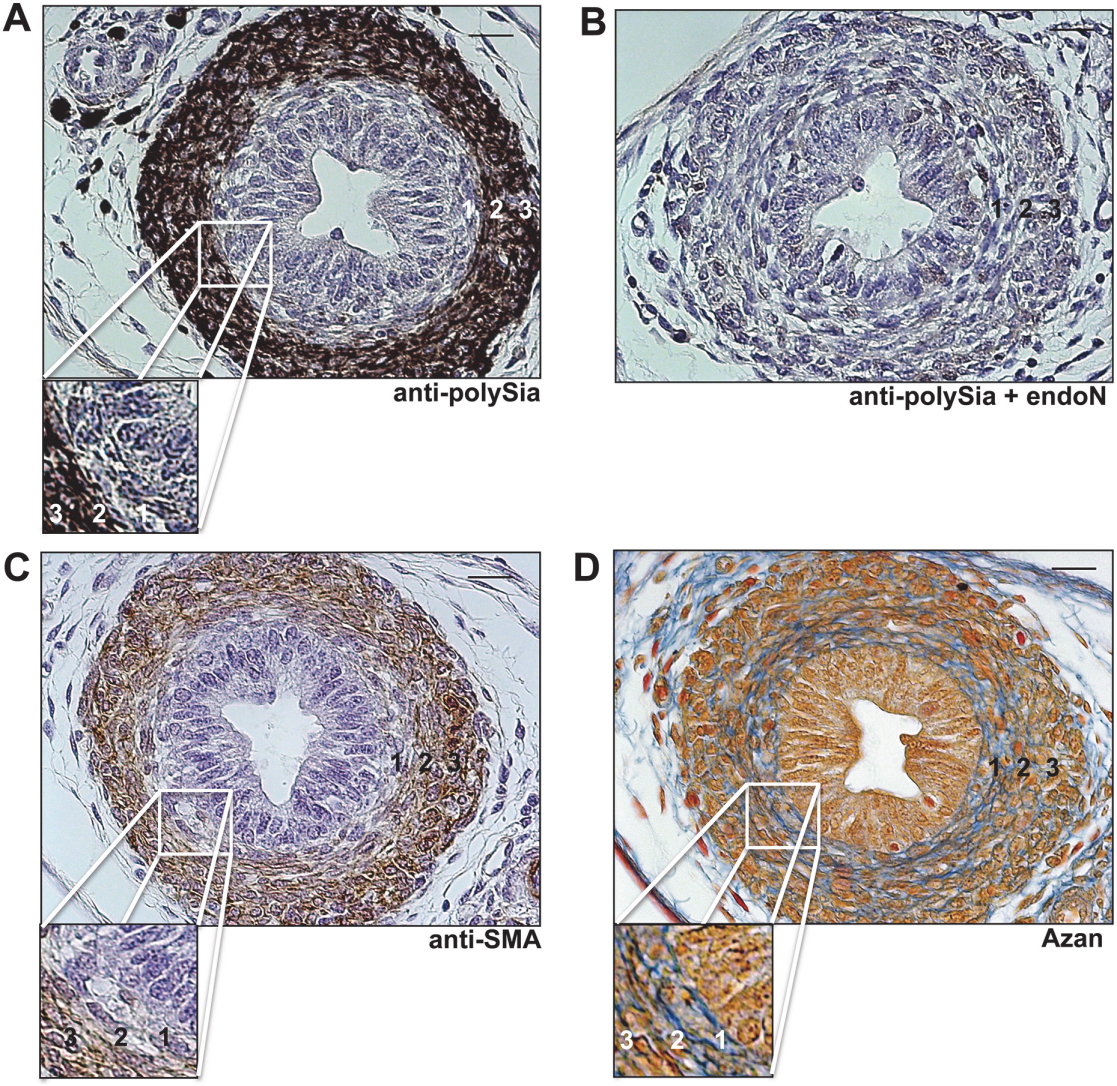
Immunohistological localization of polySia in vas deferens of postnatal mice. Transversal paraffin-embedded serial sections of vas deferens of 7 day old mice were stained with a mAb against polySia (A). For negative control, tissue sections were pretreated with endoN to degrade polySia (B). For identification of smooth muscle cells mAB against smooth muscle actin (C) were used as well as Azan staining (D) was performed. 1 = inner layer, 2 = middle layer, 3 = outer layer of smooth muscle cells surrounding the vas deferens. Cutouts show a higher magnification of the inner longitudinal layer of the muscle cells surrounding the vas deferens. Tissues (A-C) were counterstained with Haemalaun. Scale bars representing 20 μm.

PolySia was not only found in all parts of the mouse epididymal duct during postnatal development, but was also localized postnatally to smooth muscle cells surrounding the vas deferens (Fig 4A and 4B), a thick-walled duct, continuous with the epididymal duct and developing from the same embryological structure (Wolffian duct). SMA and standard azan staining verified the smooth muscle cell characteristics of polysia^+^ cells in vas deferens (Fig 4C and 4D). Heidenhain's AZAN trichrome staining revealed a higher content of collagen fibers surrounding individual cells of the inner longitudinal layer in comparison to the middle (circular) and outer (longitudinal) layer (Fig 4D). Immunostaining against polySia and SMA, however, were found to be less intensive in this inner longitudinal layer (Fig 4A and 4C).

**Fig 5:**
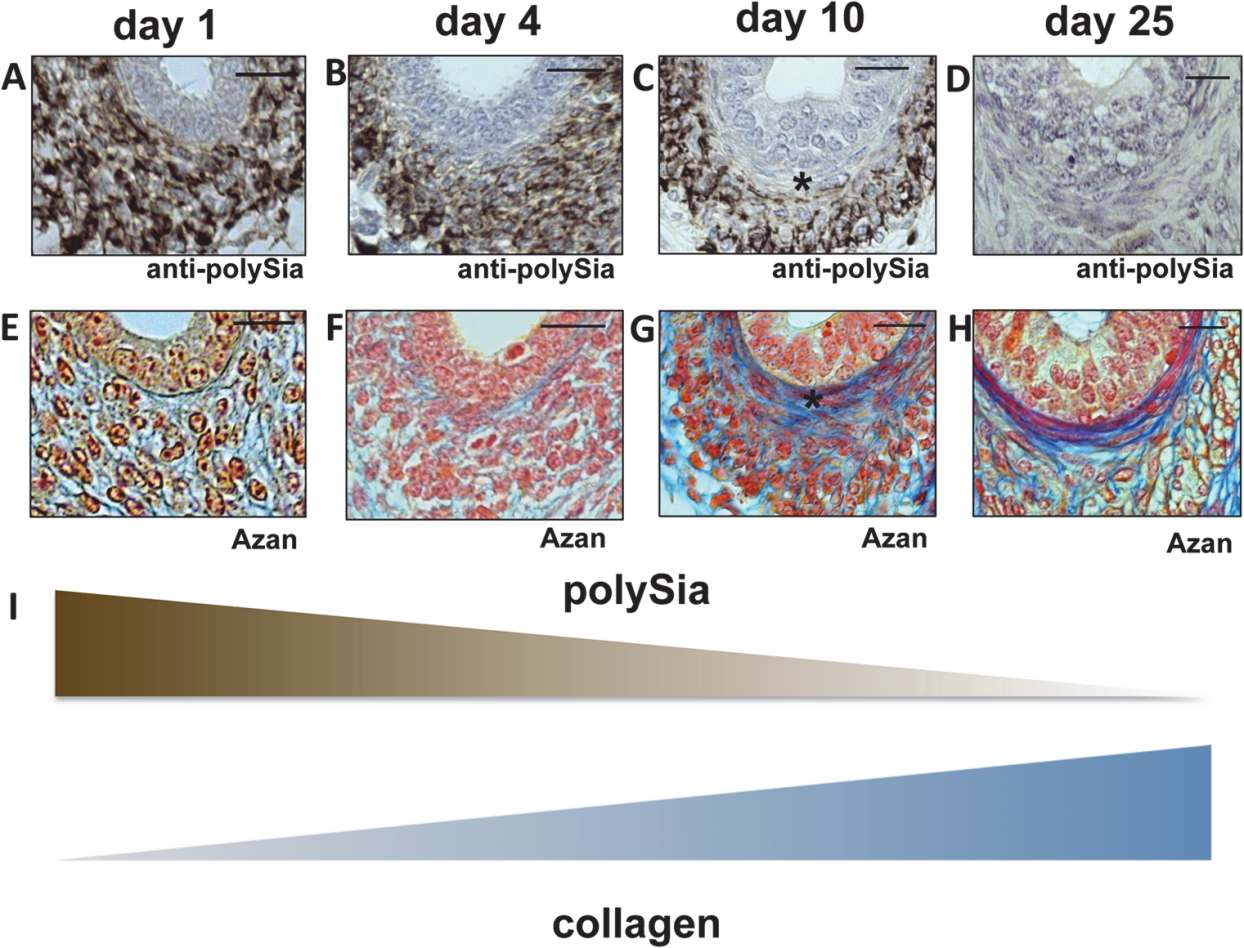
Immunohistological analysis of polySia^+^cells and collagen status of the inner longitudinal layer of smooth muscle cells in early and late postnatal mice epididymis. Paraffin-embedded serial section of epididymis (cauda) at different time points (A-H). Sections of epididymis were stained with a mAb against polySia (A-D). For negative control, tissue sections were pretreated with endoN to degrade polySia (data not shown). An anti-SMA immunostaining was used to identify smooth muscle cells (data not shown). For collagen identification Azan staining was performed (E-H). * labels same positions of smooth muscle cells. Tissues were counterstained with Haemalaun (A-D). Scale bars representing 20 μm. (I) Illustration of the inverse correlation between polySia and collagen.

In analogy to Western blot experiments (see Fig 1A) immunohistochemical investigations revealed that in epididymis polySia expression pre-dominates from postnatal day 1 to day 10, localized to smooth muscle cells at each time point, whereas it was barely detectable in these cell types at day 25 (see Fig 5 A–5 D).

Thus, smooth muscle cells are the main source of polySia-NCAM in the first 10 postnatal days in epididymis. Interestingly, similar results were observed in contractile elements of vas deferens (Fig 4) as well as in contractile elements of the oviduct during development of the postnatal female reproductive tract (data not shown).

### Proliferating smooth muscle cells express polysialylated NCAM

**Fig 6:**
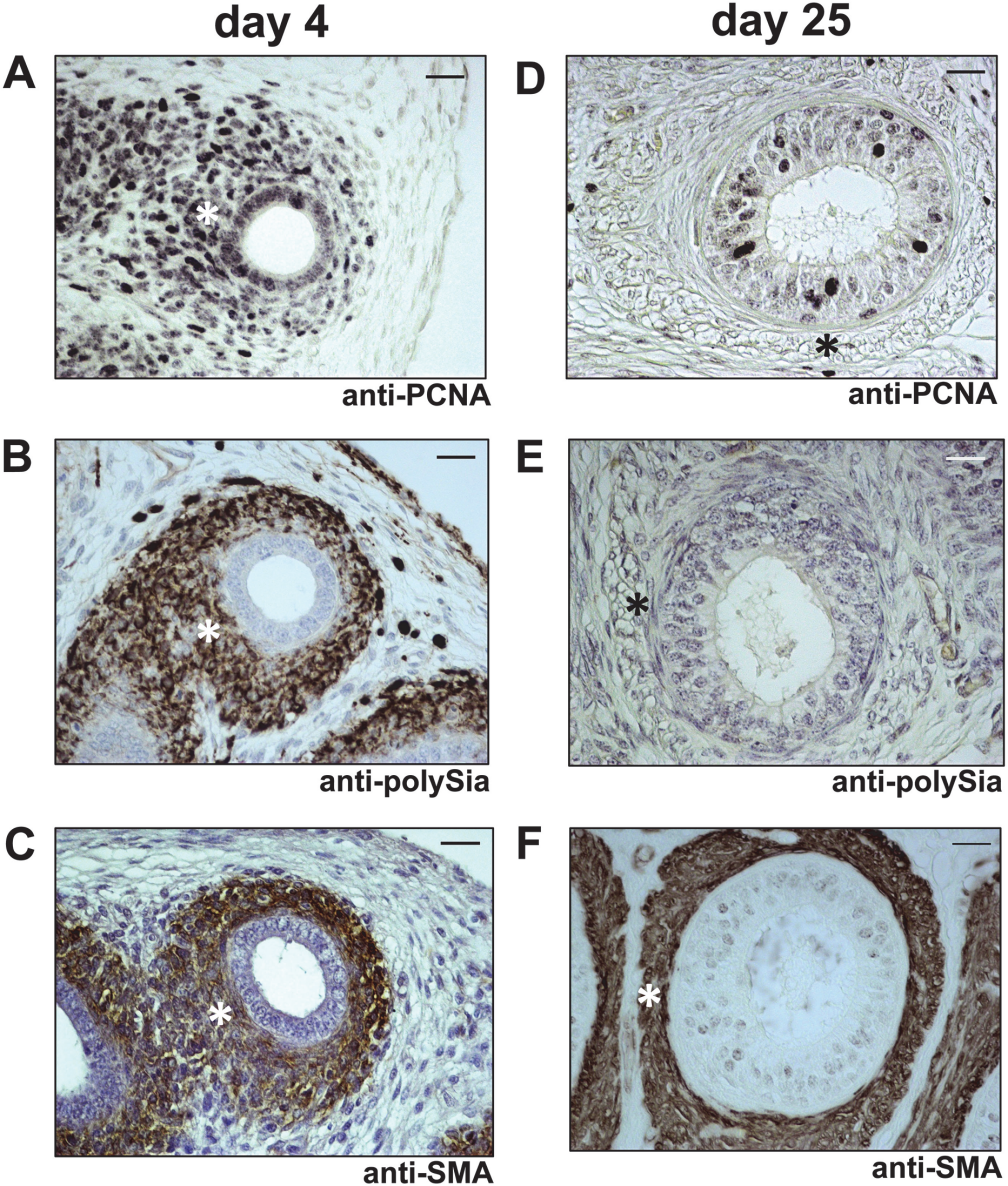
Immunohistological analysis of polySia^+^cells and cell proliferation status in early and late postnatal mice epididymis. Paraffin-embedded serial section of 4 days old (A-C) and 25 days old (D-F) mouse epididymis (cauda). Sections were stained with a mAb against proliferating cell nuclear antigen (anti-PCNA) for proliferating status (A,D) and additional stained with a mAb against polySia (B,E). For negative control, tissue sections were pretreated with endoN to degrade polySia (data not shown). For identification of smooth muscle cells a mAB against smooth muscle actin were used (C,F). * labels same positions of smooth muscle cells. Tissues were counterstained with Haemalaun. Scale bars representing 20 μm.

**Fig 7:**
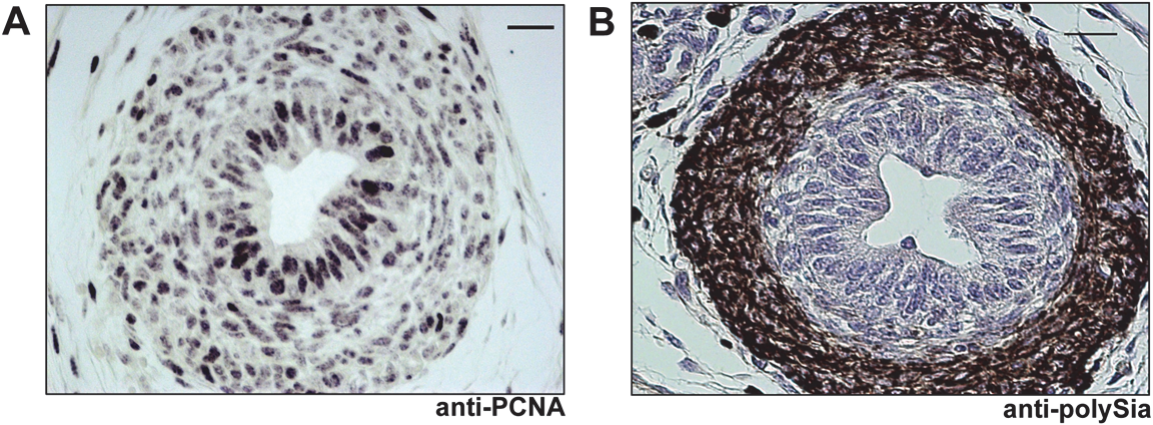
Immunohistological analysis of polySia^+^cells and cell proliferation status in postnatal vas deferens. Paraffin-embedded serial sections of 7 days old vas deferens were stained with a mAb against proliferating cell nuclear antigen (anti-PCNA) (A) and a mAb against polySia (B). For negative control tissue sections were pretreated with endoN to degrade polySia (data not shown). Tissues were counterstained with Haemalaun. Scale bars representing 20 μm.

Next, we wanted to find out whether polySia-NCAM-expressing smooth muscle cells were in the proliferation or quiescence state. Therefore, we used a cell marker against Proliferating-Cell-Nuclear-Antigen (PCNA) for proliferating cells. Since numerous smooth muscle cells showed a staining against PCNA as well as polySia during the first days after birth (see for example day 4, Fig 6 A–6 C), whereas at more advanced postnatal time points no proliferation and polysialylation of smooth muscle cells were detectable (see for example day 25, Fig 6 D–6 F), we assumed that smooth muscle cell populations prevalently express the polysialylated form of NCAM during proliferation. Similar findings of an increased polysialyltaion- and proliferation-status were observed in the vas deferens (Fig 7). Intriguingly, proliferating epithelial cells were continuously polySia negative in vas deferens.

In summary, the results demonstrated that proliferating smooth muscle cell populations of the epididymis and vas deferens are polysialylated.

### Polysialylation of smooth muscle cells decreased during the formation of collagen fibers

Interestingly, recent studies discussed novel functional relationship between extracellular matrix (ECM)-components and the balance of polysialylated and non-polysialylated NCAM during cellular events such as differentiation and migration [31]. In this context Monzo and co-workers demonstrated a collagen dependent internalization of PSA-NCAM. In order to map the presence of collagen and polySia during postnatal development AZAN staining as well as immunostaining against polySia was performed using serial sections (Fig 5). Intriguingly, in contrast to the polysialylation status of smooth muscle cells the extracellular content of collagen increased during postnatal development. These observations are especially pronounced in the area of the inner layer of smooth muscles cells surrounding the epididymal duct (Fig 5 A–5 H).

**Fig 8:**
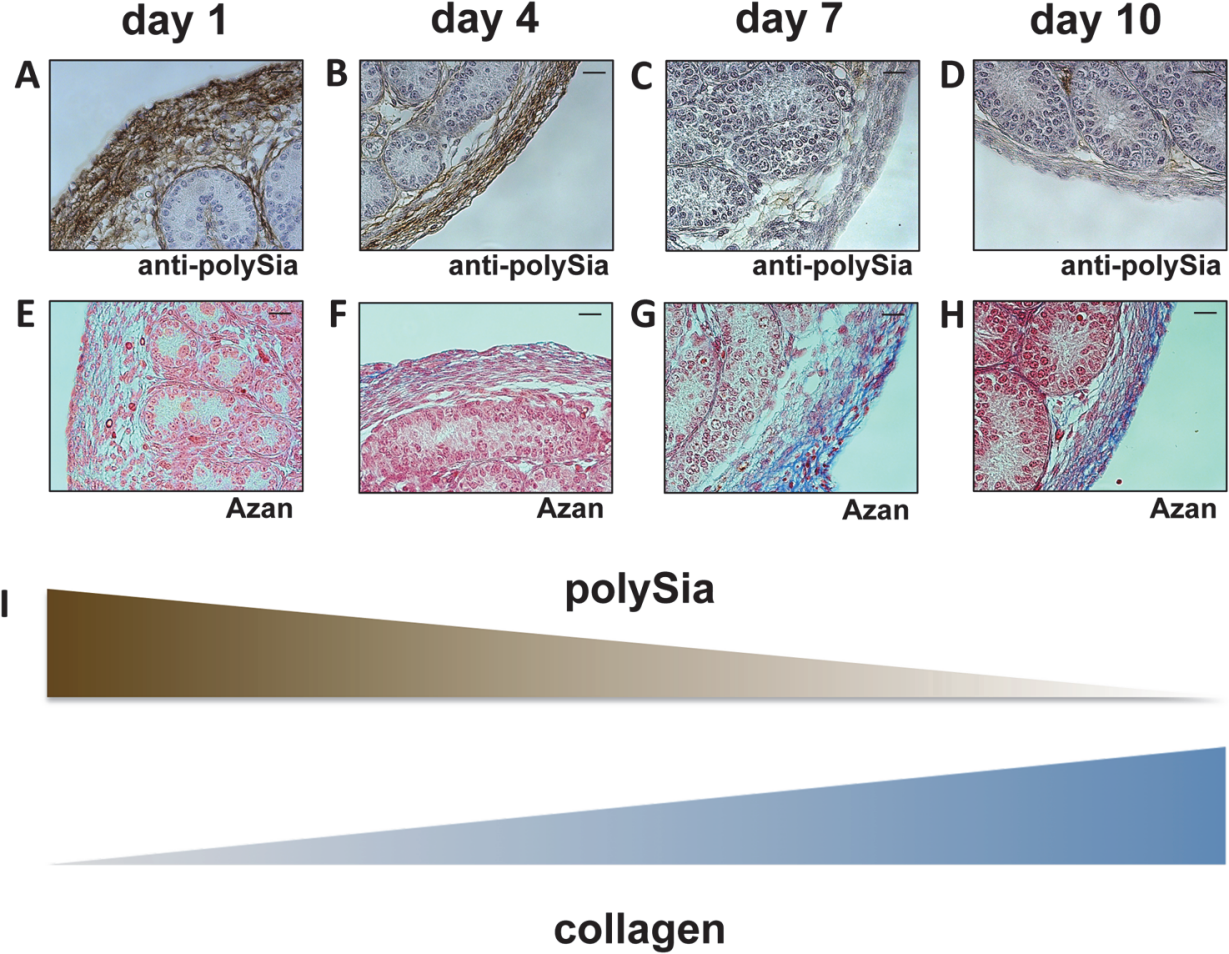
Immunohistological analysis of polySia^+^cells and collagen status of the tunica albuginea in early and late postnatal mice testis. Paraffin-embedded serial section of testis were stained with a mAb against polySia (A-D). For negative control, tissue sections were pretreated with endoN to degrade polySia (data not shown). For collagen visualization Azan staining was performed (E-H). Tissues were counterstained with Haemalaun (A-D). Scale bars representing 20 μm. (I) Illustration of the inverse correlation between polySia and collagen.

To verify the inverse correlation between polySia and collagen expression, additional investigations of the tunica albuginea in postnatal mouse testis were performed (Fig 8). The tunica albuginea represents dense connective tissue enclosing the male testicles, consisting of fibroblasts, myofibroblasts and true smooth muscle cells as well as ECM-components such as collagen fibers [32]. In the early postnatal phase a massive polysialylation of the tunica albuginea comes along with barely detectable collagenous tissue. In contrast, during postnatal development an increase of collagen positive areas was detectable whereas the content of polySia decreased (Fig 8).

Consequently, the immunohistochemical examination of postnatal testis and epididymis demonstrated that the decrease of the polysialylation status comes along with the formation of collagen fibers in smooth muscle cell populations during postnatal formation of the male reproductive tract.

## Discussion

PolySia attached to N-glycans of NCAM promotes changes in cellular interactions, proliferation as well as differentiation and plays an essential role during the development of the brain [10]. Nevertheless, also in other organs e.g. heart, lung and kidney organogenesis comes along with the expression of polysialylated NCAM [33–35]. We wanted to investigate if the polysialylated form of NCAM is also involved in the postnatal formation of the epididymis since a certain unknown NCAM isoform as well as polySia interaction partners like neurotrophins were observed in postnatal epididymis [24,25].

Therefore, murine postnatal epididymis samples were investigated for the amount of polysialylated NCAM by Western blotting, the expression levels of both polysialyltransferases by real time PCR and the localization of polySia-NCAM by immunohistochemistry. Our results demonstrated that the polysialylated form of NCAM is highly expressed in postnatal epididymis. As described for the postnatal development of the brain [7,10], polySia levels were down regulated during maturation of the epididymis. In agreement with the amount of polySia, the mRNA level of the dominant polysialyltransferase ST8SiaII was significantly reduced during postnatal formation of the epididymis.

Interestingly, smooth muscle cells were the main source of polysialylated NCAM. In adult epididymis, however, smooth muscles cells are polySia negative and only epithelial cells of the caput express polysialylated glycoproteins as recently described by our group [4]. As shown for the epididymis also smooth muscle cells of vas deferens and testis as well as the oviduct are polySia positive during development. The results let assume that polysialylation of smooth muscle cells takes in general part during postnatal development of reproductive organogenesis.

Further examination of the cell status demonstrated that proliferating smooth muscle cells prevalently express polySia-NCAM. Since smooth muscle cells are described to express receptors for neurotrophins during postnatal development of the epididymis, polySia might modulate the proliferation and/or differentiation of smooth muscle cells due to its ability to bind neurotrophins like NT-3 influencing the accessibility to their receptors. In addition, the ability of polySia, as a strongly negatively charged polymer, to inhibit interactions between cell adhesion molecules [36] resulting in a modulation of cell-cell interaction and communication as well as the remodeling of cellular surfaces might influence the cellular behavior of smooth muscle cells.

Intriguingly, with increasing extracellular concentrations of collagen, smooth muscle cells lost polysialylated NCAM. A recent study of Monzo and co-workers demonstrated that *in cellulo* collagen of the extracellular space influences the turnover of polysialylated NCAM via an induction of endocytosis [31]. Our results showed for the first time similar correlations between polySia and collagen *in vivo*. Thus, during the murine postnatal development of epididymis, vas deferens and testis the polysialylation status of smooth muscle cells may depend on the concentration of collagen reflecting the rigidity of the surrounding tissue. Nevertheless, other possible function of polysialylation cannot be excluded such as modulation of contractile potency. Interestingly, polySia staining was especially presence as long as epithelial cells are undifferentiated [37]. PolySia disappears on smooth muscle cells whereas collagen production increases, before approximately 2 weeks after birth postnatal differentiation of epithelial cells starts. This let suggest that epithelial-mesenchymal transition may influence or counteracted by polySia.

In summary, our results demonstrate that the polysialylated form of NCAM is strongly expressed by smooth muscle cells during the postnatal development of the epididymis and testis. The functional role with receptors for the neurotrophins BDNF and NT-3 let strongly suggest that a modulation of the proliferation and/or differentiation status of smooth muscle cells is regulated by these interactions as already described for neuronal cells in *in cellulo* systems [2]. Moreover, the opposing expression of polysialylated NCAM and collagen lets strongly suggest that the recently described endocytic clearance of the polysialylated form of NCAM due to an activation by collagen in the extracellular space takes place during the postnatal development of the epididymis and testis [31]. Thus, polySia might be involved in the formation of novel contractile arrangements between smooth muscle cells during the development of the male reproductive system.
